# Platelet-to-Lymphocyte Ratio and CA125 Level as a Combined Biomarker for Diagnosing Endometriosis and Predicting Pelvic Adhesion Severity

**DOI:** 10.3389/fonc.2022.896152

**Published:** 2022-06-21

**Authors:** Cuishan Guo, Chiyuan Zhang

**Affiliations:** Department of Obstetrics and Gynecology, Shengjing Hospital of China Medical University, Shenyang, China

**Keywords:** CA125, endometriosis, platelet-to-lymphocyte ratio, pelvic adhesion severity, platelet count

## Abstract

Adhesion is a significant biological characteristic of endometriosis, and accurate evaluation of the pelvic adhesion is necessary for surgical treatment. Serum CA125 is yet the most common used biomarker in the diagnosis and follow-up of patients with endometriosis despite of its high false-positive rate and low specificity. Herein, we aimed to examine the diagnostic value of the combination of the platelet-to-lymphocyte ratio (PLR) and CA125 for patients with different stages of endometriosis and their correlations with pelvic adhesion. We retrospectively analyzed the clinical data and blood count parameters of patients with both endometriosis and other benign ovarian tumors. The mean level of CA125, the PLR and the combined marker (the CA125 level multiplied by the PLR) in the EMs group were significantly higher than those in the Cyst group (P < 0.05). ROC curve analysis was used to compare the diagnostic values of serum PLR, CA125, and the combined marker in ovarian endometriosis. The cut-off value of the PLR was 176.835, with 28.3% sensitivity and 96.9% specificity. The cut-off value of CA125 was 31.67 U/mL, with 84.1% sensitivity and 87.4% specificity. The cut-off value of the combined marker was 3894.97, with 83.4% sensitivity and 95.8% specificity. It was found that the severity of adhesion in endometriosis was positively correlated with the PLR (r = 0.286, P < 0.01), CA125 (r = 0.276, P < 0.01), and combined marker (r = 0.369, P < 0.01). The combined marker showed the highest AUC value (0.751, 95% CI: 0.666–0.837), with a sensitivity of 56.0% and a specificity of 89.6%, and the cut-off value was 9056.94. Besides, the levels of CA125, PLR, and their combination were significantly elevated in patients with endometriosis. The combined marker was not only positively correlated with pelvic adhesion but also showed a greater diagnostic value and specificity than CA125 alone. These findings indicate that the combined marker may be a potential inflammatory biomarker playing an important role in the diagnosis and assessment of adhesion in endometriosis.

## Introduction

Endometriosis, a debilitating disease with features of chronic inflammation, pelvic pain, and infertility, is defined as the presence of functional endometrial glands and stroma outside the uterine cavity, which affects approximately 5%–15% of women of reproductive age (1). Endometriosis may seriously affect the quality of life of patients as well as their careers, due to its wide range of symptoms, including not only diverse kinds of pains and infertility, but also heavy menstruation, fatigue, and depression (2), resulting in a huge economic burden (3). In light of its atypical symptoms in early stage, the diagnosis and treatment of endometriosis are usually delayed up to 8–12 years (4). As the understanding of endometriosis progression continues to grow, the diagnosis of endometriosis can be made based on the comprehensive results of gynecologic examinations, hematologic laboratory tests and imaging examinations (5). Timely diagnosis, especially through non-invasive, cost-effective examinations, is essential for the early and effective treatment of endometriosis. In addition to this, however, surgical resection, to date, still a cornerstone in endometriosis management, inevitably involves a certain stage of the disease. Given that endometriosis is considered a local and systemic inflammatory response (SIR) disease with tumor-like biological behaviors, including invasion, adhesion, recurrence, and metastasis (6), the adhesion can lead to various difficulties in the surgical process (7), such as injury to the pelvic vessel, bladder, and bowel, heightening the risk of later surgical complications (8), such as bowel obstruction, chronic pelvic pain, and a high recurrence rate (9). Besides, the larger the range of ectopic endometrial lesions, the larger the area of peritoneal injury during periodic bleeding, and the greater the need for more coagulation factors (such as platelets and fibrinogen) to participate in the repairing process, leading to more extensive adhesions. Therefore, it is crucial to accurately evaluate the degree of adhesion before operative procedures, which is conducive to adequate preparation for surgery and surgical method selection, resulting in the reduction of risk of organ damage during operation and guidance of the follow-up clinical treatment.

Several hematologic parameters routinely available from routine pretreatment blood tests, such as CA125, white blood cells (WBCs), leukocyte subtypes, platelet (PLT) count, and platelet-to-lymphocyte ratio (PLR), have been investigated as cost-effective and easily obtainable circulating clinical markers in many diseases, such as pancreatitis (10), rheumatic (11) and ovarian cancer (12). Although none of the biomarkers are currently displaying enough accuracy to be used for clinical diagnosis of endometriosis, serum CA125 is yet the most common used biomarker in the diagnosis and follow-up of patients with endometriosis despite of its high false-positive rate and low specificity. CA125 is elevated in ovarian cancer, endometrial cancer, pelvic inflammatory disease, and early pregnancy. In addition, an elevated level of CA125 is mainly found in patients with advanced endometriosis, obvious pelvic inflammation, rupture of endometriotic cysts, or adenomyosis. It is of little value in the diagnosis of early-stage endometriosis. Therefore, serum CA125 is not an ideal diagnostic index.

The PLR is a simple index of the SIR that can be evaluated based on the blood parameters. The PLR can reflect the relative changes in the platelets and lymphocytes in the body, thus indirectly indicating the body’s immune disorder. Some studies have proposed that the PLR is of great significance in the development of disease and that its value can be used for the diagnosis and prognosis of systemic or local inflammatory reactive diseases and malignant tumors such as acute pancreatitis (13), breast cancer (14), lung cancer (15), and ovarian cancer (16).

In our retrospective study, we aimed to explore the role of PLR or PLR combined with CA125 as inflammatory biomarkers in endometriosis and their correlations with pelvic adhesion, to provide a new perspective for the early diagnosis, assessment of patients’ condition, formulation of intraoperative interventions, and thereby laying the foundation for the most optimal therapy of endometriosis.

## Materials and Methods

### Ethics Statement

The study was approved by the Institutional Review Board of Shengjing Hospital. We have obtained the permission of all the enrolled patients.

### Patients

A total of 145 patients with endometriosis confirmed by histopathological examination at the Shengjing Hospital of China Medical University between September 2017 and June 2020 were selected as the endometriosis group (EMs group).” The patients in the EMs group ranged in age from 18 to 50 years old. According to the revised classifications of the American Fertility Society (rAFS) staging standard established by the American Society for Reproductive Medicine, there were 7 cases with stage I, 13 cases with stage II, 70 cases with stage III, and 55 cases with stage IV endometriosis. A total of 127 patients with benign ovarian tumors other than endometriosis, as confirmed by histopathological examination during the same period in the Shengjing Hospital of China Medical University, were selected as the non-endometriosis group (Cyst group), in which tumor types included mature teratomas, serous cystadenomas, mucinous cystadenomas, simple cysts, and other benign ovarian tumors. The patients in the Cyst group ranged in age from 18 to 51 years old.

The following patients were excluded: postmenopausal patients; patients with adenomyosis or uterine fibroids as indicated by ultrasound or pathological examination; patients with acute abdomen such as torsion and rupture of adnexal mass; patients with autoimmune diseases, infectious diseases, malignant tumors, nephropathy, liver dysfunction, coagulation dysfunction, hypertension, and diabetes; and patients treated with sex hormones in the 6 months prior to hospitalization.

### Evaluation of Clinical Characteristics

The severity of pelvic adhesions was quantified according to the range and degree of adhesion, obliteration of the pouch of Douglas (POD), adhesion between bilateral fallopian tubes or between ovaries and surrounding tissues, and atresia of the fallopian tubes. The quantification system was as follows: (1) adhesion range: 1, adhesion area < 25%; 2, adhesion area = 26%–50%; and 3, adhesion area > 50%. (2) Adhesion degree: 1, loose adhesion and/or no vascular adhesion; 2, vascular adhesion and/or dense vascular adhesion; and 3, very dense adhesion and/or adhesion with no tissue interface. (3) Obliteration of the POD: 0, no obliteration; 1, partial obliteration; and 2, complete obliteration. (4) Ovarian adhesion: 0, no adhesion; 1, unilateral adhesion; and 2, bilateral adhesion. (5) Tubal adhesion: 0, no adhesion; 1, unilateral adhesion; and 2, bilateral adhesion. (6) Tubal atresia: 0, no atresia; 1, unilateral atresia; and 2, bilateral atresia. The scores for each index were added up, and the severity of adhesion was quantified according to the following: 0–1, no adhesion; 2–5, mild adhesion; 6–9, moderate adhesion; and 10–14, severe adhesion.

The degree of dysmenorrhea was graded according to the visual analog scale (VAS) system: 0, no pain; 1 to 3, mild pain (tolerable, does not affect normal life and sleep); 4 to 6, moderate pain (intolerable, the patient needs to take oral analgesics, affects sleep); 7 to 10, severe pain (patient needs intravenous analgesics, seriously affects sleep, may be accompanied by autonomic nerve dysfunction or passive position).

The uterine volume was measured by transvaginal ultrasound and calculated using the prolate ellipse equation: uterine volume = D1 * D2 * D3 * π/6, where D1, D2, and D3 represent the vertical, transverse, and anteroposterior diameters of the uterus, respectively (14). Patients’ clinical data such as age, gravidity, parity, body mass index (BMI), menstrual volume, and cyst size were obtained from electronic medical records.

### Determination of Blood Parameters

All patients had undergone routine preoperative laboratory examination during the follicular phase of the menstrual cycle within 1 month before surgery. Complete blood count parameters were measured with an automatic blood analyzer. Serum CA125 levels were measured using an electrochemiluminescence immunoassay method, and concentrations were expressed as U/mL. The PLR was defined as the absolute platelet count divided by the absolute lymphocyte count. The combined marker was obtained by multiplying the PLR by the CA125 level.

### Statistical Analysis

Statistical analysis was performed using the Statistical Package for the Social Sciences (SPSS) software package version 23.0 (SPSS Inc., USA). Continuous variables were presented as the mean ± standard error of the mean (SEM), and differences between variables were analyzed using Student’s *t*-test. Categorical variables were expressed as the number of cases and percentages (%). Differences between categorical variables were evaluated using the χ*^2^* test or Fisher exact test when necessary. The comparison of the distribution of continuous data was performed using the Kruskal-Wallis H test. Spearman analysis was conducted to determine the correlation among the measured parameters. The optimal cut-off points of each marker were evaluated by conducting receiver operating characteristic (ROC) analysis and calculating the area under the curve (AUC). A two-sided value of P < 0.05 was considered statistically significant.

## Results

### Characteristics of Patients and Concentrations of Each Biomarker in the EMs and Cyst Groups

There was no significant difference in age, BMI, gravidity times, or parity times between the EMs and Cyst groups (P > 0.05). The mean level of lymphocytes in the EMs group was significantly lower than that in the Cyst group (P < 0.01), while the mean level of CA125 and the PLR in the EMs group were significantly higher than those in the Cyst group (P < 0.05). The mean level of the combined marker was 14439.79 ± 28688.56 in the EMs group and was significantly higher in the EMs group than in the Cyst group (P < 0.01). However, there was no significant difference in WBC, neutrophil, monocyte, or platelet counts between the two groups (P > 0.05). (**Table 1**)

**Table 1:** Comparison of Characteristics Between the EMs group and Cyst group.

Variable	EMs group (n = 145)	Cyst group (n = 127)	P value
**Age (year)**	31.89 ± 7.45	30.04 ± 8.70	.060
**BMI (kg/m2)**	21.01 ± 2.38	20.70 ± 1.67	.205
**Gravidity (n)**	0.96 ± 1.18	1.11 ± 0.78	.207
**Parity (n)**	0.46 ± 0.61	0.58 ± 0.65	.116
WBC, *10^9^/L	5.89 ± 1.72	6.05 ± 1.61	.448
Neutrophil, *10^9^/L	3.53 ± 1.49	3.44 ± 1.15	.552
Lymphocyte, *10^9^/L	1.85 ± 0.58	2.07 ± 0.73	.005^*^
Monocyte, *10^9^/L	0.37 ± 0.12	0.40 ± 0.14	.059
Platelet, *10^9^/L	251.89 ± 58.34	239.83 ± 54.65	.080
**PLR**	148.29 ± 54.85	124.29 ± 34.05	.000^*^
**CA125 U/ML**	87.97 ± 115.61	19.96 ± 10.91	.000^*^
**Combined marker**	14439.79 ± 28688.56	2466.88 ± 1528.56	.000^*^
**Stage, n (%)**			
**I**	7 (4.82%)		
**II**	13 (8.97%)		
**III**	70 (48.28%)		
**IV**	55 (37.93%)		

EMs group, endometriosis group; Cyst group, non-endometriosis group; WBC, white blood cell; PLR, platelet-to-lymphocyte ratio; Combined marker, the CA125 level multiplied by the PLR.

^*^P <.05 was considered statistically significant.

### Diagnostic Value of Each Biomarker in Ovarian Endometriosis

**Figure 1:**
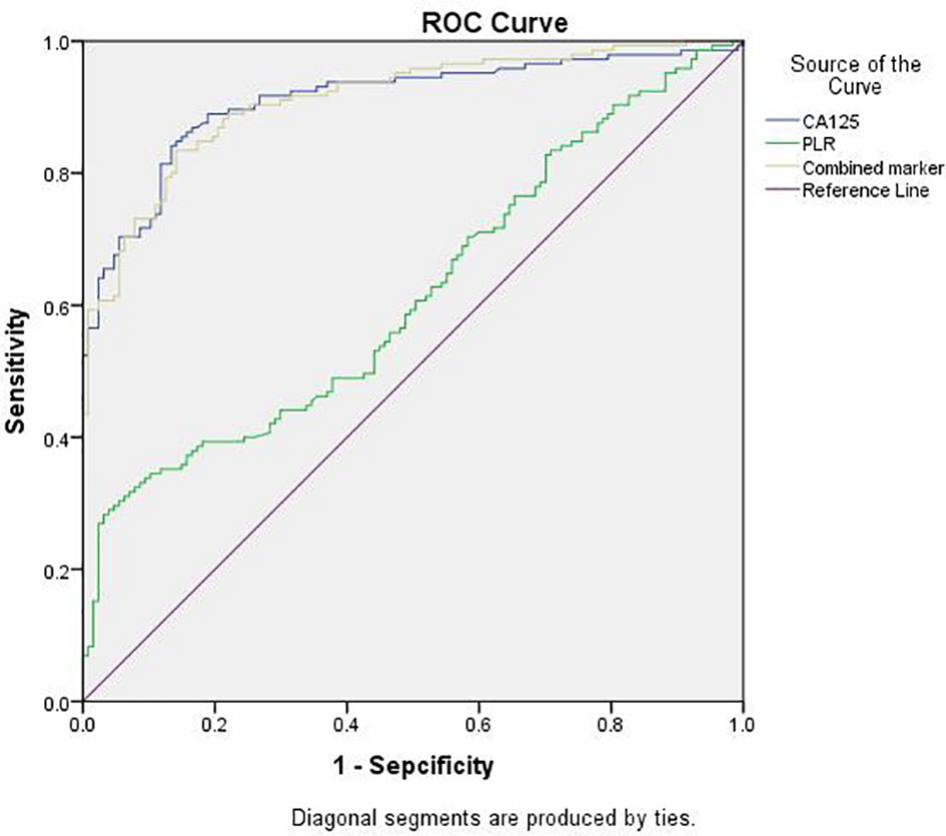
ROC curves of CA125, PLR, and the combined marker for discriminating between EMs group and Cyst group. EMs group, endometriosis group; Cyst group, non-endometriosis group; PLR, platelet-to-lymphocyte ratio; Combined marker, the CA125 level multiplied by the PLR; ROC, receiver-operating characteristic.

ROC curve analysis was used to compare the diagnostic values of serum PLR, CA125, and the combined marker in ovarian endometriosis (**Figure 1**). The AUCs of the PLR, CA125, and combined marker were 0.617, 0.910, and 0.911, respectively. The cut-off value of the PLR was 176.835, with 28.3% sensitivity and 96.9% specificity. The cut-off value of CA125 was 31.67 U/mL, with 84.1% sensitivity and 87.4% specificity. The cut-off value of the combined marker was 3894.97, with 83.4% sensitivity and 95.8% specificity (**Table 2**).

**Table 2:** Sensitivity and Specificity of CA125, PLR, and Combined Marker for Detection of Ovarian Endometriosis.

	AUC (95% CI)	Sensitivity (%)	Sepcificity (%)	Cutoff value	P value
**PLR**	0.617 (0.551-0.946)	28.3	96.9	176.835	.001^*^
**CA125**	0.910 (0.875-0.946)	84.1	87.4	31.67	.000^*^
**Combined marker**	0.911 (0.877-0.945)	83.4	95.8	3894.97	.000^*^

CI, confidence interval; PLR, platelet-to-lymphocyte ratio; Combined marker, the CA125 level multiplied by the PLR.

^*^P <.05 was considered statistically significant.

### Comparison of the None-to-Mild and Moderate - to - Severe Adhesion Groups in Terms of the Biomarkers and Patient Characteristics

In order to avoid reducing the accuracy of our analysis by examining adhesions caused by previous operations, patients with endometriosis who had a previous history of pelvic or abdominal surgery were excluded from our study. In total, 22 patients were excluded, including those with a history of cesarean section, ectopic pregnancy, or appendix surgery. Finally, 123 patients with ovarian endometriosis were further analyzed.

All patients were graded according to the method described above. There were 15 cases without adhesion, 35 cases with mild adhesion, 53 cases with moderate adhesion, and 20 cases with severe adhesion. The incidence of adhesion was 87.8%. Patients with scores of 0–5 were regarded as those belonging to the none-to-mild adhesion group, and patients with scores of 6–14 were regarded as those belonging to the moderate-to-severe adhesion group. There were 48 and 75 cases in each group, respectively.

The sociodemographic, clinical, and laboratory characteristics of the patients in each group are shown in **Table 3**. There was no significant difference in age, BMI, gravidity time, parity time, dysmenorrhea, menstrual capacity, volume of the uterus, or size of the largest endometrioma between the groups (P > 0.05). The mean levels of the PLR, CA125, and combined marker were all significantly higher in the moderate-to-severe adhesion group than in the none-to-mild adhesion group (P < 0.05). It was also found that the severity of adhesion was positively correlated with the PLR (r = 0.286, P < 0.01), CA125 (r = 0.276, P < 0.01), and combined marker (r = 0.369, P < 0.01).

**Table 3:** Comparison of characteristics between the none-to-mild adhesion group and moderate-to-severe adhesion group.

	None-to-mild adhesion group (n = 48)	Moderate-to-severe adhesion group (n = 75)	P value
**Age(year)**	29.60 ± 6.70	31.40 ± 7.35	.174
**BMI (kg/m2)**	20.83 ± 2.39	20.95 ± 2.54	.794
**Gravidity(n)**	0.73 ± 0.94	0.88 ± 1.29	.456
**Parity(n)**	0.31 ± 0.51	0.41 ± 0.62	.328
**Dysmenorrhea,n(%)**	**.913**		
**No**	13 (27.1%)	24 (32.0%)	
**Minimal**	22 (45.8%)	28 (37.3%)	
**Moderate**	6 (12.5%)	11 (14.7%)	
**Severe**	7 (14.6%)	12 (16.0%)	
**Menstrual capacity,n(%)**	**.343**		
**Normal**	46 (95.8%)	67 (89.3%)	
**Menorrhagia**	2 (4.2%)	8 (10.7%)	
**Volume of uterus, cm3**	73.04 ± 30.92	71.33 ± 35.74	.786
**Lateralization, n (%)**			
**Right**	19 (39.6%)	27 (36.0%)	
**Left**	20 (41.7%)	21 (28.0%)	.484
**Bilateral**	9 (18.7%)	27 (36.0%)	.040^*^
**Size of the largest endometrioma,cm**	5.74 ± 2.28	5.76 ± 1.91	.948
**CA125, U/ML**	53.74 ± 42.97	104.49 ± 129.90	.002^*^
**PLR**	119.57 ± 31.42	148.65 ± 56.33	.000^*^
**Combined marker**	6449.32 ± 6133.26	17364.00 ± 34610.98	.009^*^

BMI, body mass index; PLR, platelet-to-lymphocyte ratio; Combined marker, the CA125 level multiplied by the PLR.

^*^P <.05 was considered statistically significant.

The prevalence of bilateral ovarian cysts was significantly higher in the moderate-to-severe adhesion group than in the none-to-mild adhesion group (P = 0.040). There was no significant difference in the left and right lateralization between the two groups (P = 0.484).

### ROC Analysis of the Ability of the PLR, CA125, and Combined Marker to Predict the Severity of Pelvic Adhesions

**Figure 2:**
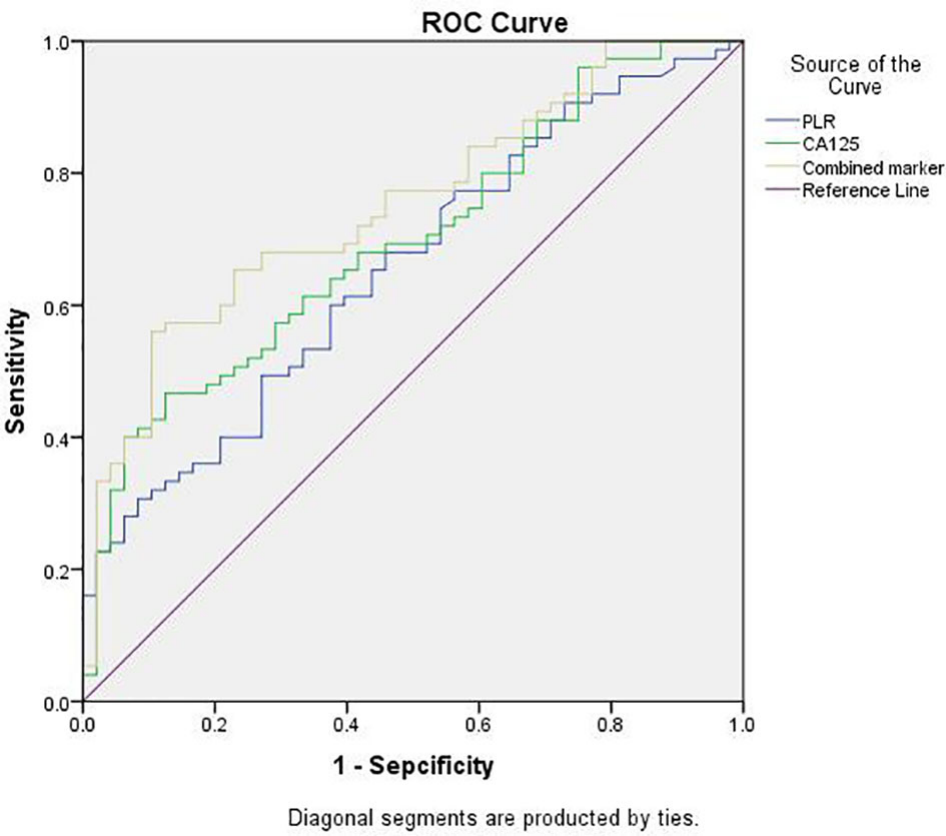
ROC analysis of PLR, CA125 and combined marker for predicting severity of pelvic adhesions. PLR, platelet-to-lymphocyte ratio; Combined marker, the CA125 level multiplied by the PLR; ROC, receiver-operating characteristic.

The optimal cut-off points for PLR, CA125, and combined marker with regard to the prediction of the severity of pelvic adhesions were further evaluated by ROC analysis (**Figure 2**). The AUC for the PLR was 0.656 (95% CI: 0.560–0.753), with a sensitivity of 60.0% and a specificity of 62.5%, and the cut-off value for the PLR was 121.83. The AUC for CA125 was 0.698 (95% CI: 0.606–0.790), with a sensitivity of 46.7% and a specificity of 87.5%, and the cut-off value for CA125 was 75.76. The AUC for the combined marker was the highest, at 0.751 (95% CI: 0.666–0.837), with a sensitivity of 56.0% and a specificity of 89.6%, and the cut-off value of the combined marker was 9056.94 (**Table 4**).

**Table 4:** Sensitivity and Specificity of the PLR, CA125, and Combined marker to Predict the Severity of Pelvic Adhesions.

	AUC (95% CI)	Sensitivity (%)	Sepcificity (%)	Cutoff value	P value
**PLR**	0.656 (0.560-0.753)	60.0	62.5	121.83	.004
**CA125**	0.698 (0.606-0.790)	46.7	87.5	75.76	.000
**Combined marker**	0.751 (0.666-0.837)	56.0	89.6	9056.94	.000

CI, confidence interval; PLR, platelet-to-lymphocyte ratio; Combined marker, the CA125 level multiplied by the PLR.

^*^P <.05 was considered statistically significant.

## Discussion

Endometriosis is a heterogeneous disease occurring in any part of the body, most commonly in the pelvic cavity (17), divided into three phenotypes involving superficial peritoneal lesions, ovarian endometriosis, and deep infiltrating endometriosis. According to the American Society for Reproductive Medicine (ASRM) classification, endometriosis is classified into four clinical stages (I, II, III and IV) based on intraoperative evaluation involving assessment of the size, location characteristics of ectopic lesions, the severity of lesions and the extent of adhesions (18, 19). Thus it can be seen that adhesions play a crucial role in the onset and progression of endometriosis, which will also be important in understanding biological behavior of ectopic lesions. Basing on the well-recognized main mechanistic hypothesis of retrograde menstruation, implantation, growth, survival and invasion of functional endometrial glands and stroma through the retrograde pathway from the fallopian tube to the peritoneal cavity can cause a series of damage to the whole body. The adhesions triggered by periodic bleeding, aseptic inflammation, and subsequent fibrous tissue hyperplasia of ectopic lesions persist throughout the above process, and can be gradually aggravated as disease progressed.

It is well known that endometriosis is an aseptic and estrogen-dependent chronic inflammatory disease. The levels of inflammatory factors (like CRP, IL-6, IL-8, and TNF-a) in the peripheral blood and peritoneal fluid of patients with endometriosis are increased (20, 21). Inflammatory factors stimulate monocytes and T lymphocytes to produce selective chemokines, which induce ectopic endometrial adhesion, implantation, and angiogenesis (22). These cytokines and chemokines are related to cascade reaction and immune function impairment. The growth of ectopic endometrial lesions not only recruits large numbers of macrophages, monocytes, and other inflammatory factors, but also leads to peritoneal trauma and fibrin formation, leading to peritoneal remodeling and subsequent formulation of adhesions (23).

However, the evidence for the degree of adhesions and the severity of symptoms is inconsistent and even contradictory because of high symptom heterogeneity of endometriosis. Patients with few adhesions may have more pain or infertility, whereas patients with severe adhesions may have few symptoms. Not only this, but severe adhesions can cause diverse organ damage, involving peritoneal injury and fibrosis, vascular injury, intestinal injury, bladder damage, ureteral injury, and postoperative relevant complications, such as infection, rectovaginal fistula, bladder, and bowel dysfunction (24). Similarly, it is worth considering whether adhesions are related to endometriosis recurrence. High staging and insufficient thoroughness of primary surgery are both high-risk factors of recurrence in patients with endometriosis (9, 25), which are closely associated with adhesions, as the magnitude of adhesions is proportional to the staging of endometriosis. Concurrently, severe adhesions impede complete endometriotic lesion removal, which becomes the major bottleneck, resulting in incomplete surgical resection, and are more likely to relapse. Therefore, adhesions may take an essential part in the recurrence of endometriosis. There is currently no consensus on timing of endometriosis surgery, despite once during the entire endometriosis life should be stressed, ideally. Therefore, it is extremely important for surgeons to perform a preoperative assessment of adhesions, which contributes to making the decision of further imaging investigations, such as magnetic resonance imaging (MRI), and preoperative bowel preparation. In addition, preoperative informed consent about adhesions and subsequent potential risk of surgery can benefit from it. Notably, it is critical to develop individualized surgical plans.

Nevertheless, no single existing examination enables a complete assessment of pelvic adhesions prior to operation. With the development of research on the pathogenesis of endometriosis, the correlation between the occurrence and development of endometriosis and the inflammatory response has been widely recognized. In particular, neutrophils, lymphocytes, monocytes, and the PLR can be used as indicators to evaluate the degree of SIR. Yavuzcan et al. (26). found no significant difference in the PLR between the stage 3/4 endometriosis group and the control group; that is, no diagnostic value was identified that indicates advanced-stage endometriosis. However, Yang et al. (27). retrospectively analyzed the diagnostic value of the PLR in 197 patients with moderate-to-severe endometriosis and found that the mean absolute values of serum CA125, the PLR, and the combination value (the value of CA125 multiplied by the value of the PLR) were significantly increased for patients in the moderate-to-severe endometriosis group. The AUCs for CA125, the PLR, and the combined marker were 0.943, 0.587, and 0.929, respectively. Serum CA125 was shown to be better than the PLR and the combined marker for diagnosing moderate-to-severe endometriosis. Additionally, studies have shown that a level of CA125 higher than 65 U/mL helps to identify high-risk patients with severe pelvic adhesions for whom preoperative bowel preparation should be performed, with a sensitivity of 76%, specificity of 71%, positive predictive value of 76%, and negative predictive value of 93.2% (28).

In our research, on one hand, the levels of CA125, the PLR, and the combined marker in the moderate-to-severe adhesion group were significantly higher than those in the none-to-mild adhesion group, suggesting they were positively correlated with the severity of pelvic adhesions. Not only that, but ROC analysis was performed to assess the utility of these parameters for predicting the severity of pelvic adhesions. As a result, the combined marker had the highest AUC value, while the PLR was the most sensitive (89.6%) biomarker, indicating that the combined marker may play a valuable role in predicting the severity of pelvic adhesions prior to operation. From another perspective, in light of heterogeneity and complexity of endometriosis, no matter assessment or management, before operation, we could easily obtain the preliminary assessment of endometriosis adhesions by a highly efficacious and cost-effective haematological parameter. Subsequently, we could risk stratify patients for management decision-making, including not only further MRI imaging examination, bowel preparation, but also accurate assessment of patients’ condition, more targeted consent fully informed, appropriate preoperative preparation, intraoperative injury prevention, and management of postoperative complications. On the other hand, in the present study, the levels of the PLR, CA125, and the combined marker in the EMs group were significantly higher than those in the Cyst group. Although the level of PLR was higher in the patients with endometriosis, its diagnostic value was still lower than that of CA125. The diagnostic value and specificity of the combined marker were higher than those of CA125 alone, while the sensitivity of the combined marker was similar to that of CA125, indicating that the combined marker is more valuable for diagnosing endometriosis. This finding seems to report contradictory results, whereas the prior study only focused on patients with advanced-stage endometriosis, while our study analyzed patients across all stages of endometriosis. Although none of the biomarkers have been well recognized better for early diagnosis of endometriosis, the combined marker still shows higher specificity and sensitivity, compared with CA125, indicating good validity. In the future, the combined marker may help to provide an initial indication of studies of endometriosis diagnosis performed on larger populations. Taken together, our results implicate the combined marker of PLR and CA125 as a clinical potential biomarker of endometriosis, in support of early diagnosis and preoperative evaluation of adhesions.

However, our study has a few limitations. First, as a retrospective study, our study naturally had a large bias. Our sample size was small and from a single institution; further research involving a larger and more diverse sample size is required to confirm the clinical utility of our findings. Moreover, the retrospective nature of the study did not allow us to evaluate values of other systemic inflammation markers, such as CRP, interleukins, and procalcitonin. Finally, in this study, we only studied endometriosis without further stratified analysis for phenotypes.

In conclusion, the present study demonstrated that the level of the combined PLR and CA125 marker was significantly higher in patients with endometriosis than those with other benign ovarian tumors. The diagnostic value and specificity of the combined marker were higher than those of CA125 alone, while the sensitivity was similar, indicating that the combined marker is more effective for diagnosing endometriosis than CA125 alone. Meanwhile, the combined marker was positively correlated with the severity of pelvic adhesion, suggesting its potential prediction in the degree of pelvic adhesion in patients with endometriosis, providing a meaningful reference for making better-informed decisions on further imaging investigations, adequate preoperative bowel preparation, detailed preoperative informed consent, prediction of intraoperative or postoperative risks and individualized surgical plans developing.

## Data Availability Statement

The raw data supporting the conclusions of this article will be made available by the authors, without undue reservation.

## Ethics Statement

The studies involving human participants were reviewed and approved by The Institutional Review Board of Shengjing Hospital. The patients/participants provided their written informed consent to participate in this study.

## Author Contributions

CZ conceived the project and collected the data. CG analyzed the data and wrote the manuscript. All authors contributed to the article and approved the submitted version.

## Funding

This work was supported by the New Technology and New Project of China Medical University under Grant No. 3110210722.

## Conflict of Interest

The authors declare that the research was conducted in the absence of any commercial or financial relationships that could be construed as a potential conflict of interest.

## Publisher’s Note

All claims expressed in this article are solely those of the authors and do not necessarily represent those of their affiliated organizations, or those of the publisher, the editors and the reviewers. Any product that may be evaluated in this article, or claim that may be made by its manufacturer, is not guaranteed or endorsed by the publisher.
